# Descemet membrane endothelial keratoplasty in patients after radiation therapy for uveal melanoma

**DOI:** 10.1186/s12886-026-04787-9

**Published:** 2026-04-02

**Authors:** Anna-Karina B. Maier, Dhoksina Papa, Aline Riechardt, Jens Heufelder, Oliver Zeitz, Antonia M. Joussen, Tina Dietrich-Ntoukas

**Affiliations:** 1 Department of Ophthalmology, Charité – Universitätsmedizin Berlin, Corporate Member of Freie Universität Berlin, Humboldt- Universität zu Berlin and Berlin Institute of Health, Berlin, Germany; 2 BerlinProtonen am Helmholtz-Zentrum Berlin für Materialien und Energie, Charité-Universitätsmedizin Berlin, Berlin, Germany

**Keywords:** DMEK, Uveal melanoma, Proton beam therapy, Brachytherapy

## Abstract

**Purpose:**

To evaluate the visual outcomes and postoperative complications after Descemet membrane endothelial keratoplasty (DMEK) in patients after radiation therapy for uveal melanoma.

**Methods:**

In this retrospective observational study, 9 eyes of 9 patients after radiation therapy for uveal melanoma who received DMEK surgery at the Charité – Universitätsmedizin Berlin were included. Preoperative patients` characteristics were analyzed. Postoperative results including visual acuity and endothelial cell density and complications were evaluated.

**Results:**

Best-corrected visual acuity improved 3, 12 and 24 months after DMEK (preoperative: 0.88 ± 0.47 logMAR, after 3 months: 0.52 ± 0.30 (*p* = 0.049), after 12 months: 0.60 ± 0.35 logMAR (*p* = 0.66), after 24 months: 0.60 ± 0.45 logMAR (*p* = 0.357)) compared to preoperatively. Endothelial cell density decreased 3 and 12 months after DMEK (preoperative: 2327 ± 242 cells/mm^2^, after 3 months: 1831 ± 384 cells/mm^2^ (*p* = 0.068), after 12 months: 1350 ± 996 cells/mm^2^ (*p* = 0.180)). Re-bubbling was performed in 55.6% of eyes after DMEK. One patient developed a postoperative macular edema, and another one a distant metastasis of the liver 20 years after last tumor treatment. Two-year-incidence of graft rejection was 0%, of graft failure 25.0% (95%KI -17.5%, 67.5%), of IOP-elevation 55.6% (95% KI 12.1%, 99.1%) and of post-DMEK glaucoma 16.7% (95%KI -13.1%, 46.5%).

**Conclusions:**

Our results confirm that DMEK surgery is feasible and improves the visual acuity in patients with local control of uveal melanoma after radiation therapy. However, the complications rate is high - including graft failure, postoperative IOP elevation and post-DMEK glaucoma - and complications of the radiation treatment limit the achievable visual acuity.

**Supplementary Information:**

The online version contains supplementary material available at 10.1186/s12886-026-04787-9.

## Introduction

Uveal melanoma is the most common primary intraocular tumor in adults with an age adjusted risk of 5 per 1 million [1]. It includes melanoma of the iris, choroid and ciliary body. Resection, radiation therapy, and enucleation are the first-line treatment options for uveal melanomas currently. Radiation therapy includes brachytherapy with episcleral plaques (e.g., ^125^I or ^106^Ru), teletherapy with heavy charged particles (proton beam therapy, helium ion therapy), and stereotactic radiotherapy. As radiation therapies for the treatment of uveal melanoma improved in recent years and allow an eye preservation, other vision-impairing eye diseases, that occur coincidently, or vision-impairing complications of the treatment play a more important role in the postoperative course [2]. Once local tumor control has been achieved, treatment of these vision-impairing eye diseases and complications can be considered. This also includes corneal endothelial disorders like Fuchs endothelial corneal dystrophy (FECD), bullous keratopathy or graft failure after previous keratoplasty. These diseases can be treated very successfully by Descemet membrane endothelial keratoplasty (DMEK), because it enables a fast visual rehabilitation, reduction of corneal edema and reduction of corneal thickness and bullae [3–7]. Because only an isolated Descemet membrane and its endothelium are transplanted, a near-normal anatomic corneal restoration is possible. The excellent results of visual acuity in the literature suggest that it is also an option for the treatment of posterior corneal endothelial disorders in patients after radiation therapy for uveal melanoma [2]. Because many different complications as result of the uveal melanoma and its treatment such as secondary glaucoma, corneal neuropathy, corneal decompensation, limbal stem cell failure, radiation retinopathy and radiation optic neuropathy, retinal detachment and maculopathy can occur, DMEK in those patients may be more advanced and results may be less favourable [8–13].

Therefore, we investigated the pre-, intra- and postoperative results of DMEK surgery in patients after radiation therapy for uveal melanoma.

## Materials and methods

### Patients

In this retrospective observational study, we enrolled eyes from patients after radiation therapy for uveal melanoma, that underwent DMEK surgery at the Department of Ophthalmology, Charité – Universitätsmedizin Berlin, Campus Virchow Klinikum, between January 2013 and December 2020 performed by three experienced surgeons (N.T., A.-K. M., T.D-N.) after screening of 2188 DMEK surgeries performed during this time period. This study adhered to the ethical standards of the Declaration of Helsinki. Institutional ethical approval was obtained by the Ethics Committee of the Charité – Universitätsmedizin Berlin (EA4/167/16) and (EA2/108/12). For this retrospective, single-center study formal consent was not required; the Ethikkommission, Charité – Universitätsmedizin Berlin approved the waiver of consent. The patients gave their consent to the surgery in the usual manner after being informed about the surgical procedure.

### Preoperative and postoperative evaluation

Examinations were performed preoperatively and one, three, 12 and 24 months after DMEK surgery, including a complete ophthalmologic evaluation containing objective refraction using an auto refractometer (RM-8900 Auto Kerato-Refractometer, Topcon Corporation, Itabashi-ku, Tokyo, Japan), best corrected visual acuity (BCVA) tested with a Snellen chart, endothelial cell density (NIDEK CEM-530, specular microscope, NIDEK Co., LTD, Gamagori, Aichi, Japan), slit-lamp examination and central corneal thickness (CCT) (NIDEK CEM-530, specular microscope, NIDEK Co., LTD, Gamagori, Aichi, Japan).

### Graft and surgical technique

All patients underwent single DMEK procedure with a graft with minimum endothelial cell density of 2000 cells/mm^2^. The graft and surgical technique and the postoperative regime, especially the re-bubbling, have been described in detail in previous studies [14]. The graft diameter was routinely chosen between 8.0 and 8.5 mm, depending on donor age. No graft from a pseudophakic donor was used. Definitions of IOP-elevation, post-DMEK glaucoma, graft rejection and graft failure were used as defined in detail by Maier AB et al. [15].

### Statistical methods

Normality was tested for all outcome measures and the appropriate statistical test was used. Descriptive statistics were expressed as median and range or mean ± standard deviation (SD). We used Kaplan-Meier survival analysis to estimate incidences for graft rejection, graft failure, IOP-elevation and post-DMEK glaucoma. Differences were considered statistically significant when P values were less than 0.05.

## Results

After screening of 2188 DMEK surgeries performed between January 2013 and December 2020, 9 eyes from 9 patients were included in this retrospective study. In these eyes uveal melanoma was diagnosed and treated before DMEK surgery. Data of the included patients (6 female and 3 male) were summarized in Table 1 and supplement Table 1. All included patients were pseudophakic. Apart from the indication of FED for DMEK surgery, two patients underwent surgery due to bullous keratopathy. In these two cases, multiple prior ocular surgical procedures had been performed: in one case due to secondary glaucoma, and in another case due to hypotony of the eye following radiation therapy. In a third case, graft failure after penetrating keratoplasty was observed in the context of repeated rejection episodes. Two patients with pre-existing secondary glaucoma were treated by trabeculectomy with mitomycin C before DMEK and in one case with additional cyclophotocoagulation. One of these two patients needed an additional glaucoma surgery, cyclophotocoagulation, after DMEK surgery to control intraocular pressure (IOP).

**Table 1:** Preoperative data of included patients

Age (years)	73.1 ± 12.3
Tumor localization	
- Iris	*n = 5 (55.6%)*
- Choroid	*n = 4 (44.4%)*
Tumor treatment	
- Brachytherapy with episcleral plaque ^106^Ru	*n = 4 (44.4%)*
- Proton beam therapy	*n = 4 (44.4%)*
- Both	*n = 1 (11.1%)*
- additional transpupillary thermotherapy (TTT)	*n = 2 (22.2%)*
Time between first radiation therapy and DMEK (months)	117.9 ± 84.0 (range 19.5–229.6)
Reason for DMEK surgery	
- FED	*n = 6 (66.7%)*
- Bullous keratopathy	*n = 2 (22.2%)*
- Graft failure after perforating keratoplasty	*n = 1 (11.1%)*
Visual acuity (LogMAR)	0.88 ± 0.47
Preoperative glaucoma diagnosis	*n* = 2 (22.2%)
Preoperative pars-plana vitrectomy	*n* = 3 (33.3%)
Endothelial cell density of graft (cells/mm^2^)	2291 ± 227
Age of donor (years)	74.1 ± 13.3
Sex of female & male donors	3 female and 6 male

### Visual acuity and endothelial cell density

Visual acuity improved 3, 12 and 24 months after DMEK (preoperative: 0.88 ± 0.47 logMAR, after 3 months: 0.52 ± 0.30 (*p* = 0.049), after 12 months: 0.60 ± 0.35 logMAR (*p* = 0.66), after 24 months: 0.60 ± 0.45 logMAR (*p* = 0.357)) compared to preoperatively. The factors limiting visual acuity were as follows: three patients presented with epiretinal gliosis; one patient exhibited recurrent macular edema in addition to advanced glaucoma; another patient had advanced glaucoma; and one patient demonstrated persistent choroidal folds following hypotony of the eye.

Endothelial cell density of the graft decreased 3 and 12 months after DMEK (endothelial cell density of the graft: 2291 ± 227 cells/mm^2^, after 3 months: 1831 ± 384 cells/mm^2^ (*p* = 0.068), after 12 months: 1350 ± 996 cells/mm^2^ (*p* = 0.180)).

Due to missing data on central corneal thickness, statistical analysis was not performed for this parameter.

### Complications

Additional application of intracameral air, so called re-bubbling, was performed in 55.6% of eyes after DMEK, once in 4 eyes and twice in one eye. One patient showed a postoperative macular edema, which disappeared after a few months following a temporary intensified topical steroid therapy. This patient had epiretinal gliosis as a risk factor for macular edema.

No patient developed local recurrence of the tumor during the follow-up after DMEK. One patient developed a distant metastasis of the liver 18 months after Re-DMEK, 5 years and 9 months after first DMEK, performed because of a graft failure after penetrating keratoplasty, and 23 years and 9 months after ruthenium-applicator brachytherapy of choroidal melanoma of the iris, and 20 years after proton beam therapy of melanoma recurrence.

Two-year-incidence of graft rejection was 0%, of graft failure 25.0% (95% KI -17.5%, 67.5%), of IOP-elevation 55.6% (95% KI 12.1%, 99.1%) and post-DMEK glaucoma 16.7% (95% KI -13.1%, 46.5%).

No donor tissue was lost during DMEK preparation in this study.

## Discussion

Our results show that DMEK surgery improves the visual acuity in patients with local control of uveal melanoma after radiation therapy, but results are limited by other complications after radiation treatment. The two-year incidences of graft failure, postoperative IOP elevation and post-DMEK glaucoma are high.

In our study, the proportion of patients with localization of the melanoma in the iris was greater than in the choroid, although iris melanoma is the most uncommon of all uveal melanomas (2% versus 90% choroidal) [16]. As described by Riechardt et al., the risk of a corneal decompensation and necessity of a keratoplasty is high in patients with a proton beam therapy of the whole anterior segment treating iris melanoma [2]. However, the most common reason for DMEK in our study was the coincidence of FED and uveal melanoma and not corneal decompensation due to radiation. It is also possible that patients with corneal decompensation after radiation treatment of uveal melanoma were not even considered for DMEK because they had other vision-limiting diseases or an involvement of other corneal layers, so that a penetrating keratoplasty seemed more appropriate, or because local tumor control was not achieved. The results of mean visual acuity show an improvement after 3, 12 and 24 months after DMEK surgery, but these remain significantly lower than those normally achieved after DMEK [3, 5–7]. Only two patients showed a visual acuity of 0.1 logMAR in the postoperative course, in all others visual acuity improved, but stayed limited. This is due to multiple complications like radiation optic neuropathy, glaucomatous optic neuropathy, radiation retinopathy, retinal detachment and maculopathy, but also corneal changes from dry eye syndrome to limbal stem cell failure, that occur as result of the uveal melanoma or of the radiation treatment of the tumor [10–13, 17], which also limit the results concerning visual acuity. Macular OCT was not performed in a fully standardized manner at all follow-up visits, which represents a limitation. However, available OCT examinations revealed that several patients exhibited epiretinal gliosis, in some cases associated with transient cystoid changes and in one case with clinically significant macular edema requiring temporary intensification of topical steroid therapy. These findings underline the increased inflammatory potential of irradiated eyes and may further explain the limited long-term visual acuity despite successful graft attachment and corneal clarity.

All included eyes were pseudophakic, which excludes lens status as a confounding factor for postoperative visual acuity outcomes. This homogeneity allows a more direct interpretation of the corneal contribution to visual rehabilitation in this cohort. Furthermore, no donor graft from a pseudophakic donor was used, and donor characteristics were comparable without notable abnormalities, minimizing potential donor-related bias.

Two-year-incidences of graft failure, IOP elevation and post-DMEK glaucoma are higher in these patients than usual in patients after DMEK [3–7, 14]. Here, the multiple complications of the tumor and its radiation therapy play also a role. Cohen et al. showed that a complicated anterior segment - including previous glaucoma surgery, post-penetrating keratoplasty, peripheral anterior synechia, previous pars-plana vitrectomy, aniridia, aphakia, anterior chamber intraocular lens and iris-fixated intraocular lens - increases risk for graft failure [18]. This is also represented in our study collective with patients presenting with previous glaucoma surgery, after pars-plana vitrectomy and after penetrating keratoplasty. For IOP elevation and post-DMEK glaucoma, it has been also shown that a pre-existing glaucoma and the diagnosis bullous keratopathy and graft failure (versus FED) increased the risk for these complications [14]. Additionally, the risk of graft failure is higher in eyes with pre-existing glaucoma, especially in patients with glaucoma diagnosis classified as all others except primary open-angle / primary angle-closure glaucoma and pseudoexfoliative glaucoma [14, 15, 19, 20]. Secondary glaucoma in patients with uveal melanoma after radiation therapy is probably more difficult to treat, has a higher risk for complications and shows additional structural changes of the anterior chamber probably influencing the re-bubbling rate and higher inflammatory response increasing the risk of developing postoperative graft failure [12, 21]. In our experience, consistent treatment of IOP elevation is particularly necessary, while no adjustment to the standard regime after DMEK was necessary with regard to anti-inflammatory and lubrication therapy.

Re-bubbling rate was higher in our study collective compared to re-bubbling rates using the same treatment strategies [14, 15]. Here, as mentioned for the other complications above, the underlying diagnoses such as post-graft failure or pre-existing glaucoma, but also structural changes of the anterior chamber and tissues due to the tumor and its treatment resulting in changes of the posterior corneal surface may play a role [22, 23].

In none of the patients a local tumor recurrence occurred after DMEK and none of the patients presented with inoculation metastasis as a consequence of the DMEK. However, one patient developed distant metastases 20 years after the last tumor treatment. Since this is repeatedly reported in the literature, regular semi-annular liver ultrasound examinations are recommended until the end of life, as distant metastases typically occur there first [24–26].

The main limitations were the small sample size and the retrospective character due to the rare coincidence of DMEK and local tumor control after radiation therapy of uveal melanoma. Additionally, treatment therapies to achieve local tumor control of uveal melanomas and complications of the treatment differ between the included patients. However, this corresponds to the real-world data available for the treatment of uveal melanoma. Corneal sensitivity was not systematically assessed in our cohort. Given that radiation therapy may affect corneal innervation and ocular surface stability, this parameter could be relevant for graft survival and epithelial integrity and therefore visual acuity.

Our results confirm that DMEK surgery is feasible and improves the visual acuity in patients with local control of uveal melanoma after radiation therapy. However, the complication rate, especially graft failure, postoperative IOP elevation and post-DMEK glaucoma, is high and other complications of the radiation treatment limit the achievable results concerning visual acuity. We initially approached DMEK in this subgroup with caution due to the pre-existing ocular comorbidities and the potentially limited visual prognosis. However, the consistent postoperative functional benefit observed in most patients suggests, that an early DMEK should be considered in carefully selected patients if indicated and if local tumor control had been achieved. However, this approach requires thorough preoperative counseling regarding the elevated perioperative and postoperative risk profile and is recommended in specialized centers and under appropriate close monitoring, particularly with attention to IOP control.

## Supplementary Information

Below is the link to the electronic supplementary material.

Supplementary Material 1

## Data Availability

All data generated or analysed during this study are included in this published article and its supplementary information files.
