# Supplementary material for: Descemet membrane endothelial keratoplasty in patients after radiation therapy for uveal melanoma
Source: BMC Ophthalmol. 2026 Apr 2;26:161. doi: 10.1186/s12886-026-04787-9 (PMC13047794; doi:10.1186/s12886-026-04787-9)
Supplement: Supplementary file 1 — Supplementary Material 1 [file 12886_2026_4787_MOESM1_ESM.docx]

| **Indication for radiation** | **Tumor treatment** | **Irradiated corneal volume [%]** | **Tumor dose PBT [CGE]** | **Scleral contact dose RU-106 [Gy]** | **Indication for DMEK** |
| --- | --- | --- | --- | --- | --- |
| Iris melanoma | PBT | 63% | 50 |  | FED |
| Iris melanoma | PBT | 62% | 50 |  | FED |
| Iris melanoma | PBT | 59% | 50 |  | FED |
| Iris melanoma (resection)/ recurrence (PBT) | PBT | 100% | 50 |  | Corneal decompensation |
| Iris melanoma (RU-106)/Local recurrence (PBT) | RU-106 + PBT | 100% | 50 | no data available | Transplant decompensation after perforating keratoplasty and re-KPL |
| Choroideal melanoma | RU-106 | -- |  | 420 | FED |
| Choroideal melanoma | RU-106 + TTT | -- |  | 500 | Corneal decompensation after bulbus hypotonia after various interventions |
| Choroideal melanoma | RU-106 | -- |  | no data available | FED |
| Choroideal melanoma | RU-106 + TTT | -- |  | no data available | FED |

Supplement table 1: Tumor characteristics and data of treatment protocol
